# Prevalence, Antimicrobial Resistance, and Diversity of *Salmonella* along the Pig Production Chain in Southern Brazil

**DOI:** 10.3390/pathogens8040204

**Published:** 2019-10-24

**Authors:** Luciano dos Santos Bersot, Valéria Quintana Cavicchioli, Cibeli Viana, Raquel Cristina Konrad Burin, Anderson Carlos Camargo, José Paes de Almeida Nogueira Pinto, Luís Augusto Nero, Maria Teresa Destro

**Affiliations:** 1 Departamento de Ciência Veterinárias, Universidade Federal do Paraná, Setor Palotina, Palotina, PR 85950-000, Brazil; cibeli_viana@hotmail.com; 2 Departamento de Veterinária, Universidade Federal de Viçosa, Viçosa, MG 36570-900, Brazil; valeriacavic@gmail.com (V.Q.C.); raquel.burin@hotmail.com (R.C.K.B.); anderson.c.camargo@hotmail.com (A.C.C.); nero@ufv.br (L.A.N.); 3 Departamento de Higiene Veterinária e Saúde Pública, Universidade Estadual Paulista, Botucatu, SP 18618-000, Brazil; jose.paes@unesp.br; 4 Faculdade de Ciências Farmacêuticas, Universidade de São Paulo, São Paulo, SP 05508-900, Brazil; mariateresa.destro@biomerieux.com

**Keywords:** *Salmonella*, pig production chain, PFGE, contamination routes, resistance

## Abstract

Control of *Salmonella* spp. in food production chains is very important to ensure safe foods and minimize the risks of foodborne disease occurrence. This study aimed to identify the prevalence and main contamination sources of *Salmonella* spp. in a pig production chain in southern Brazil. Six lots of piglets produced at different farms were tracked until their slaughter, and samples were subjected to *Salmonella* spp. detection. The obtained isolates were serotyped, subjected to antimicrobial resistance testing, and pulsed field gel electrophoresis (PFGE). *Salmonella* spp. was detected in 160 (10.2%) samples, and not detected in pig carcasses after final washing or chilling. Among the 210 *Salmonella* spp. isolates, *S.* Typhimurium was the most prevalent (n = 101) and resistant to at least one antimicrobial. High resistance rates were detected against tetracycline (83.8%), chloramphenicol (54.3%), and trimethoprim-sulfamethoxazole (33.3%). The isolates that were non-susceptible to three or more classes of antimicrobials (n = 60) were considered multidrug-resistant (MDR), and isolates resistant to up to six of the tested antimicrobials were found. PFGE allowed the identification of genetic diversity and demonstrated that farm environment and feed supply may be sources for the dissemination of *Salmonella* spp. along the production chain. The results revealed the sources of *Salmonella* contamination in the pig production chain and highlighted the risks of antimicrobial resistance spread.

## 1. Introduction

Pork is the second most widely animal protein consumed worldwide, and Brazil is the fourth world producer and exporter [1]. The southern Brazilian region has a concentration of more than 60% of pig production, according to data from the Brazilian Association of Animal Protein [2]. Pork meat exportation has increased in Brazil since 2000, leading to modifications across the industries to fit the international requirements from the World Trade Organization regarding safety and quality [3]. Based on safety aspects, the control of pathogens such as *Salmonella* spp. is mandatory to ensure safe foods to consumers and minimize the risks of the occurrence of foodborne disease. Ensuring food quality and safety is very important mainly due to the changing food habits of the population, the popularization of mass feeding establishments, and the globalization of food supply [4,5,6,7].

*Salmonella* spp. can be considered as a component of pig intestinal microbiota [5]. They are usually asymptomatic carriers, excreting the pathogen intermittently or when stressed [8]. Nowadays, pig farms usually breed animals in high density barns, facilitating the spread and persistence of *Salmonella* spp. in the environment [6,9]. Many countries are concerned with regard to the spread and persistence of multidrug-resistant (MDR) *Salmonella*, and these strains have been reported in pigs, slaughterhouses, and the final products as well as in clinical samples [10,11,12,13,14]. The use of antibiotics in different steps of pig production can contribute to the spread of *Salmonella* resistant strains [10]. Genes related to resistance against antimicrobials are often transferred through mobile elements (particularly plasmids) amongst bacteria including *Salmonella*, which makes the fight against antimicrobial resistance a challenge [15,16].

*Salmonella* spp. is an important foodborne pathogen in Brazil, and according to data presented by the Brazilian Ministry of Health, this pathogen was responsible for more than 11% of the recorded and investigated foodborne diseases in the country between 2009 and 2018. Pork products were considered relevant foods associated with these cases/outbreaks [17], but these data are considered to be underestimated in Brazil. Data from the United States and European Union also indicate *Salmonella* spp. as the main pathogen related to foodborne diseases caused by the ingestion of contaminated meat products including pork [18,19].

The identification of *Salmonella* spp. contamination sources along the pig production chain is a key point for taking preventive and corrective measures. The proper characterization of *Salmonella* spp. isolates and the identification of the main contamination routes as well as the spread of multidrug-resistant strains can subsidize the development of reliable programs to control and limit the spread of antimicrobial resistance. Considering these aspects, the present study aimed to determine the prevalence of *Salmonella* spp. along the pig production chain in Paraná State (southern Brazil), determine the antimicrobial resistance profiles, and possible contamination routes within this meat production chain. 

## 2. Results and Discussion

Pig production is one of the most important husbandry activities in Brazil, therefore studies focusing on the detection of pathogens are important to ensure sanitary control during the production and safety of the meat distributed for sale. The occurrence of *Salmonella* spp. in the pig production chain evaluated in the present study is presented according to the site and type of sample in Table 1. The results showed that in the nursery (maternity and piglets barns), only 4.8% (32/662) of samples were positive for *Salmonella*, while in the pig finishing farms, the prevalence increased to 13.9% (88/636). The overall prevalence of *Salmonella* at the slaughterhouse was 14.8% (40/270), which was detected mainly from feces (21/25); all carcasses after end washing and chilling tested negative (Table 1). The presence of the *Salmonella* in the feed supply certainly contributes to pig contamination and consequently persistence in the barn environment. High discharge of *Salmonella* in feces collected at the slaughterhouse confirm the constant re-introduction of the pathogen in this environment. 

The higher frequencies of *Salmonella* spp. in the pig finishing farms when compared to the nursery can be explained by two key factors: (1) maternal immunity is transferred from sows to their piglets via colostrum and milk, which contributes to the development of a protecting microbiota against pathogens during this period [20]; and (2) in the finishing farms, the longer period of stay (110 days in average) [21] and close contact among animals from different production farms facilitates the contamination and leads to the high excretion of *Salmonella* spp due to stress conditions. In agreement with our findings, a study conducted in Brazil by Kich et al. [22] demonstrated higher frequencies of *Salmonella* spp. carrier animals in finishing farms when compared to the previous production steps. Stress conditions (observed during transport and before slaughter) may contribute to the major discharge of the pathogen at the slaughterhouse [8,23].

The presence of *Salmonella* spp. in pig jowls may be explained by the high pathogen excretion in feces, associated with the habit of coprophagy [21,24]. *Salmonella* spp. in the pig oral cavity can often lead to contamination of the jowls as well as its presence in the animal’s intestine leads to contamination in the mesenteric lymph nodes. Several studies have demonstrated that the incision of lymphatic tissues can significantly affect bacterial contamination on the pig carcass [25,26]. In the present study, *Salmonella* spp. was not identified among the 50 carcasses sampled after final washing and chilling, demonstrating that procedures adopted during slaughter ensured the microbiological safety of the carcasses (Table 1). Focusing on microbial hazards such as *Salmonella*, the official guidelines for swine slaughtering in Brazil have changed, being more based in a risk analysis approach [27]. 

A total of 210 *Salmonella* spp. isolates obtained from different sources along the pig production chain were subjected to serological identification (Table 2). Serotypes Typhimurium, Mbandaka, Panama, Derby, and Agona were the most prevalent, similar to those observed in another study conducted in the southern region of Brazil (Kich, Coldebella, Morés, Nogueira, Cardoso, Fratamico, Call, Fedorka-Cray, and Luchansky [22]. *S.* Typhimurium was the most common serotype isolated in both farms and slaughterhouses (Table 2); this serotype has already been described as the most frequent in pig production worldwide [28,29,30,31], and is most associated with human salmonellosis outbreaks in the European Union [18,19].

The isolates obtained from feces showed the highest serotype diversity, mainly those from piglet production and pig finishing farms (data not shown). Interestingly, various serotypes were isolates from both farms and slaughterhouses, while *S*. Give, *S*. Meleagridis, and *S*. Worthington were isolated only from feces samples from animals in the slaughterhouse (Table 2), suggesting that contamination occurred during transportation, since animal transport is relevant for the spread of *Salmonella* spp. [32,33,34].

Overall, only 17 (8.1%) of the 210 isolates were sensitive to all tested antimicrobials: resistance to tetracycline was the most common (83.8%), followed by chloramphenicol (54.3%), and trimethoprim-sulfamethoxazole (33.3%). The antimicrobial resistance profiles of the isolates, according to the number of antibiotics to which the strain was resistant, are presented in Table 3. From the 193 isolates resistant at least to one tested antibiotic, it was identified that many isolates exhibited resistance to at least three (n = 43), four (n = 12), five (n = 4), and six (n = 1) antimicrobials (Table 3). All *S.* Typhimurium (n = 101) isolates obtained in the present study were resistant to at least one tested antibiotic and 30 isolates were resistant to at least three antimicrobial agents. A total of 111 isolates from different serotypes exhibited simultaneous resistance to chloramphenicol and tetracycline. The emergence of MDR *Salmonella* is a global health concern [10,13]. Herein, simultaneous resistance to chloramphenicol, tetracycline, and trimethoprim-sulfamethoxazole was recorded in 57 isolates, representative of different serotypes, while 14 isolates were resistant to these antimicrobials plus ampicillin (Table 3). In addition, one isolate from serotype Panama was resistant to six antibiotics. Despite the efforts of the Brazilian Ministry of Agriculture to regulate the use of antimicrobials in animal production since the 1990s as a growth promoter and clinical treatment, the obtained data might be associated with the previously extensive use of these drugs in pig production [10,35]. Besides data regarding the emergence of antibiotic resistance in food production, a National Prevention and Control Program was recently established by the Brazilian Ministry of Agriculture. This program aims to promote strategic actions such as epidemiological studies, the implementation of infection prevention and other control measures to promote rational use to prevent antibiotic resistance spread [36]. Our results revealed that the situation is worrying, and further studies will allow the proper identification of molecular mechanisms associated with antimicrobial resistance exhibited by the MDR isolates recovered in the present study. 

**Figure 1:**
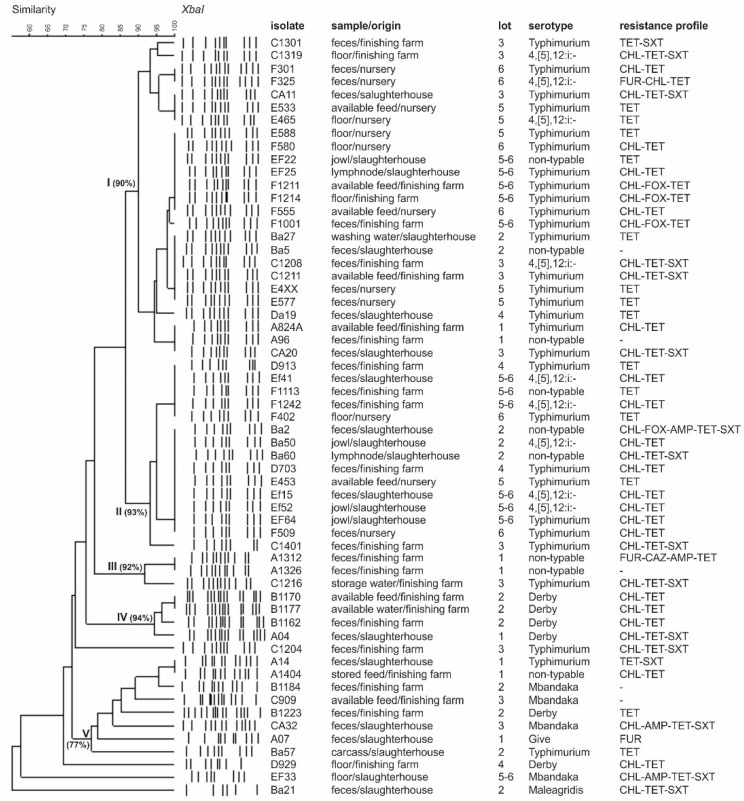
Schematic representation (genetic profile, isolate identification, isolation source, lot, serotype, and antibiotic resistance profile) of 59 *Salmonella* isolates obtained from the pork production chain in southern Brazil. Macro-restriction was conducted with *XbaI*. Identity was estimated using the Dice coefficient (5% tolerance). Lot “5-6” was composed of animals from lots 5 and 6, mixed after nursery. Antibiotics: FUR: furazolidone (15 µg), CHL: chloranphenicol (30 µg), CAZ: ceftazidime (30 µg), FOX: cefoxitin (30 µg), AMP: ampicillin (10 µg), TET: tetracycline (30 µg), SXT: trimethoprim-sulfamethoxazole (25 µg).

Based on previous results, 59 *Salmonella* spp. isolates representing positive samples sources were selected for PFGE. Considering the adopted UPGMA parameters, the isolates were grouped in five main clusters with similarities ranging from 77 to 94% (Figure 1). It was possible to identify in each cluster the isolates from different sources and pig production steps, from the nursery to the slaughter. Identical *Salmonella* profiles were also obtained from samples recovered from different lots, indicating that genetically close isolates are spread across Paraná State (Figure 1). We identified isolates belonging to serotype Typhimurium and its monophasic variant 4,5,12:i:-, which presented identical genetic profiles by PFGE, and can be explained by the fact that they are from the same serogroup and share a high genetic similarity [14]. 

The PFGE has been useful and accurate for tracking contamination sources, allowing the identification of *Salmonella* persistence, cross contamination, and distribution in swine production and pork processing [22,28,29,34,37]. Herein, the PFGE results demonstrate the possible role of the farm environment and feed supply in the dissemination of *Salmonella* spp. along the production chain (Figure 1), as revealed by previous studies [9,38]. Interestingly, isolates obtained from different nurseries and different pig finishing farms (feces and environment) and isolates from the slaughterhouse (environment, jowls, mesenteric lymph nodes) shared identical genetic profiles, as observed in clusters I and II (Figure 1). This might indicate cross-contamination or possible contamination from the same source in the nurseries, farms, or slaughterhouse.

The present study identified critical contamination points in the pig production chain in southern Brazil including feed supply, which may have contributed to the *Salmonella* introduction or maintenance in the pig barn environment. The isolates exhibiting an identical genetic profile isolated from different sources and farms revealed the spread of *Salmonella* in Parana State. In addition, *Salmonella* isolated from the same lots exhibiting different PFGE profiles demonstrated that the animals were exposed to a diversity of *Salmonella* throughout their life (Figure 1).

## 3. Materials and Methods 

### 3.1. Sampling

Six pig lots were selected in three different farms located in Paraná State, southern Brazil. The lot selection criterion enables sampling during breeding (piglet production and pig finishing) and slaughtering steps, at 10 day intervals (day 0 to day 150). The piglet lots were selected on the birth date and each lot was sampled throughout the nurseries (maternity and piglet barns), pig finishing farms (pig barns), and slaughterhouses (environment and carcasses), resulting in a total of 1568 samples. 

At pig farms, the following samples were collected: feed stored (250 g), feed available for animal consumption (250 g), water from nipple (250 mL), water from storage (250 mL), feces collected directly from the floor (80 g), and barn floor (100 cm^2^). All animals sampled in the pig finishing farms were sent to the same commercial slaughterhouse inspected by the Brazilian Ministry of Agriculture. At the slaughterhouse, the following samples were collected: clean lairage floor (100 cm^2^); feces (80 g); scalding water (250 mL); pig wash water (250 mL); evisceration table (100 cm^2^); carcass saw (100 cm^2^); carcass wash water (250 mL); carcasses after evisceration, after final washing, and after chilling, (100 cm^2^ each); pig jowls (300 g); and mesenteric lymph nodes (50 g). Surface sampling was conducted by swabbing one sterile sponge for each point (3M Microbiology, St. Paul, MN, USA), previously moistened with 10 mL of buffered peptone (1% w/v) saline (0.85% w/v) solution (BPS, Oxoid Ltd., Basingstoke, UK).

### 3.2. Salmonella spp. Detection and Serotyping 

Pre-enrichment of the samples was conducted in buffered peptone water at 1% (w/v) (BPW, Oxoid Ltd., Basingstoke, UK), which was 25 g of solid sample added to 225 mL of BPW, sponges were added to 90 mL of BPW, and 200 mL of the water samples were centrifuged at 3500× *g* for 15 min and the pellet was added to 100 mL of BPW incubated at 35 °C for 18 to 24 h. *Salmonella* isolation was conducted according to the protocol described by USDA [39]. Colonies with typical *Salmonella* characteristics on triple sugar agar and lysine iron agar (Oxoid) were subjected to a serological test by using polyvalent somatic and flagellar antisera (Probac do Brasil, São Paulo, SP, Brazil), and further biochemical characterization by the following tests: urease, indole, methyl red, Voges-Proskauer, citrate, motility, and malonate [39]. Isolates identified as *Salmonella* spp. (n = 210) were serotyped by slide agglutination (Kauffmann–White–Le Minor scheme) using diverse somatic and flagellar antisera in the Bacteriology Section of the Instituto Adolfo Lutz (São Paulo, SP, Brazil) and Enterobacteriaceae Laboratory from the Fundação Oswaldo Cruz (Rio de Janeiro, RJ, Brazil). Isolates were kept stored in trypticase soya broth (TSB, Oxoid) added to glycerol at 10% at −20 °C.

### 3.3. Antimicrobial Resistance Testing

*Salmonella* isolates (n = 210) were subjected to the disk-diffusion assay, as described by the Clinical & Laboratory Standards Institute [40]. Isolates were grown in BHI (Oxoid) at 37 °C for 24 h, the inoculum was adjusted by the McFarland standard and a swab was dipped into the adjusted suspension, then spread onto the surface of plates containing Mueller Hinton agar (Oxoid), where disks of the following antimicrobials were added: ampicillin (10 μg), cefoxitin (30 μg), chloramphenicol (30 μg), ceftazidime (30 μg), cefotaxime (30 μg), tetracycline (30 μg), imipenem (10 μg), furazolidone (15 μg), amikacin (30 μg), and trimethoprim-sulfamethoxazole (25 μg). The plates were incubated at 35 °C for 18 h. All antimicrobials were purchased from Oxoid and *Escherichia coli* ATCC 25922 was used as a pan-susceptible quality control. The diameters of the zones of inhibition were measured and the *Salmonella* isolates were characterized as resistant or susceptible according the Clinical & Laboratory Standards Institute [41]; in this study, *Salmonella* isolates that exhibited an intermediate resistance were considered as resistant. 

### 3.4. Pulsed Field Gel Electrophoresis (PFGE)

Based on *Salmonella* spp. positive results for each step of pig production, 59 isolates were selected for PFGE. Aliquots of the selected isolates were transferred to TSB (Oxoid), incubated at 37 °C overnight, and added to agarose (Bio-Rad Laboratories, Hercules, CA, USA) for plug preparation. Plugs were subjected to lysis and washed, and the obtained DNA was subjected to macrorestriction with 50 IU of *Xba*I (Promega, Madison, WI, USA) at 37 °C for 2 h, as recommended by the Centers for Disease Control and Prevention (CDC) and described by Ribot et al. [42]. Macrorestriction products were separated in agarose gels (Agarose Seakem Gold 1%, w/v, buffer TE 0.5X) in a CHEF-DR II system (Bio-Rad) following the parameters recommended by the CDC. The gels were stained with GelRed (Biotium Inc., Hayward, CA, USA) and the band patterns were analyzed by using BioNumeric 6.6 software (Applied Maths NV, Sint-Martens-Latem, Belgium). Pulse Marker 50 (1000 kb, Sigma-Aldrich Corp., St. Louis, MO, USA) was added to each gel for band normalization. Isolate similarities were calculated considering the Dice coefficient, 5% of tolerance, and the dendrogram with the genetic profiles of selected isolates was constructed considering the unweighted pairwise grouping with mathematical averaging (UPGMA) algorithm.

## 4. Conclusions

Taken together, our results revealed that serotype Typhimurium is spread along the pig production chain in Paraná State, southern Brazil, which is introduced continuously into the slaughterhouse through animal carriers. Our results showed that farms must adopt measures to control *Salmonella* spp. in the feed supply to prevent pig contamination through this foodborne pathogen. Even through *Salmonella* spp. was identified at different sampling sites including mesenteric lymph nodes and jowls, all 50 carcasses sampled after final washing and chilling tested negative for this pathogen, indicating the good manufacturing practices were properly applied in the slaughterhouse. However, the high rate of MDR strains isolated in the pig production chain highlight that implementation measures are needed to prevent the spread of antibiotic resistance in the environment. Monitoring antimicrobial resistance in the pork production chain is very relevant as resistant strains can be transferred to humans. Our study emphasizes the high genotypic diversity of *Salmonella* and the importance of the swine environment as a reservoir of MDR *Salmonella*. 

## Figures and Tables

**Table 1:** Detection of *Salmonella* spp. along the pig production chain in Paraná State, southern Brazil.

Site	Site Detail	Type of Sample	Sample Size	n	*Salmonella* spp. Positive Samples (%)
nursery	maternity	feed (available)	250 g	45	0
		feed (stored)	250 g	54	1 (1.8)
		water (available)	250 mL	51	0
		water (stored)	250 mL	18	0
		floor	100 cm² *	54	0
		feces	80 g	63	1 (1.6)
	piglets barn	feed (available)	250 g	69	7 (6.1)
		feed (stored)	250 g	69	1 (0.8)
		water (available)	250 mL	69	0
		water (stored)	250 mL	23	0
		floor	100 cm²	69	10 (8.1)
		feces	80 g	78	12 (8.5)
pig finishing farm	pig barn	feed (available)	250 g	99	18 (18.2)
		feed (stored)	250 g	117	2 (1.7)
		water (available)	250 mL	117	1 (0.8)
		water (stored)	250 mL	38	1 (2.6)
		floor	100 cm²	117	18 (15.4)
		feces	80 g	148	48 (35.1)
slaughterhouse	environment	clean lairage floor	100 cm²	25	4 (16.0)
		feces	80 g	25	21 (84.0)
		scalding water	250 mL	5	0
		pigs washing water	250 mL	5	0
		evisceration table	100 cm²	15	4 (26.7)
		carcasses saw	100 cm²	15	0
		carcasses washing water	250 mL	5	1 (20.0)
	pig carcass	after evisceration	100 cm²	30	1 (6.7)
		after final washing	100 cm²	50	0
		backbone	100 cm²	15	0
		after chilling	100 cm²	50	0
		pig jowls	3 carcasses, 300 g	15	6 (40.0)
		mesenteric lymph nodes	3 carcasses, 50 g	15	3 (20.0)
Total				1568	160 (10.2)

* surface samples obtained by swabbing with sterile sponges moistened with 10 mL of buffered peptone water at 1% (w/v) (Oxoid Ltd., Basingstoke, UK).

**Table 2:** *Salmonella* serotypes identified in 210 isolates obtained from the pig production chain in Paraná State, southern Brazil.

Serotype	Farms	Slaughterhouse	Total
Typhimurium	86	15	101
Mbandaka	12	9	21
Panama	11	08	19
Derby	18	0	18
Agona	10	5	15
Give	0	3	3
Worthington	0	1	1
Meleagridis	0	1	1
Poona	1	0	1
Non identified			
non-typeable	8	5	13
Typhimurium monophasic variant 4,5,12:i:-	6	4	10
Typhimurium monophasic variant 4,5,12:-:1,2	4	0	4
rough	3	0	3
Total	159	51	210

**Table 3:** Antimicrobial resistance profile of *Salmonella* isolates recovered along the pig production chain in southern Brazil. All isolates that exhibited resistance against antimicrobials from three or more classes were considered as multidrug-resistant.

n ATB	Resistance Profile *	n Isolates	Serotypes
6	AMK-FUR-CHL-AMP-TET-SXT	1	Panama
5	CHL-FOX-AMP-TET-SXT	2	Panama, non-typeable
	FUR-CHL-AMP-TET-SXT	2	Mbandaka, Panama
4	CHL-AMP-TET-SXT	9	Mbandaka
	FUR-CHL-TET-SXT	1	Typhimurium
	AMK-FUR-CHL-TET	1	non-typeable
	FUR-CAZ-AMP-TET	1	non-typeable
3	CHL-TET-SXT	42	Derby, non-typeable, Meleagridis, Typhimurium
	CHL-FOX-TET	1	Typhimurium
2	CHL-TET	51	Derby, non-typeable, Typhimurium, rough strain
	CHL-SXT	3	Mbandaka
	TET-SXT	4	Typhimurium, Agona
	AMK-TET	2	Typhimurium
	CTX-TET	2	Derby, Typhimurium
	CTX-SXT	1	Worthington
	FUR-SXT	1	Agona
	FUR-TET	2	Typhimurium
1	TET	55	Derby, non-typeable, Panama, Typhimurium, rough
	FUR	5	Agona, Give, Mbandaka
	STX	4	Derby, Mbandaka
	AMK	2	Mbandaka, Poona
	CHL	1	Mbandaka
0	none	17	Agona, Derby, Give, Mbandaka, Panama, Worthington, non-typeable

* AMP: ampicillin (10 μg), FOX: cefoxitin (30 μg), CHL: chloramphenicol (30 μg), CAZ: ceftazidime (30 μg), CTX: cefotaxime (30 μg), IPM: imipenem (10 μg), FUR: furazolidone (15 μg), AMK: amikacin (30 μg), SXT: trimethoprim-sulfamethoxazole (25 μg).
